# The PI3K inhibitor taselisib overcomes letrozole resistance in a breast cancer model expressing aromatase

**DOI:** 10.18632/genesandcancer.100

**Published:** 2016-03

**Authors:** Klaus P. Hoeflich, Jane Guan, Kyle A. Edgar, Carol O'Brien, Heidi Savage, Timothy R. Wilson, Richard M. Neve, Lori S. Friedman, Jeffrey J. Wallin

**Affiliations:** ^1^ Department of Translational Oncology, Genentech, Inc., South San Francisco, CA, USA; ^2^ Department of Oncology Biomarker Development, Genentech, Inc., South San Francisco, CA, USA; ^3^ Department of Molecular Biology, Genentech, Inc., South San Francisco, CA, USA

**Keywords:** PI3K, Letrozole, Breast Cancer, GDC-0032, Akt

## Abstract

Letrozole is a commonly used treatment option for metastatic hormone receptor-positive (HR+) breast cancer, but many patients ultimately relapse. Due to the importance of phosphoinositide-3 kinase (PI3K) in breast cancer, PI3K inhibitors such as taselisib are attractive for combination with endocrine therapies such as letrozole. Taselisib was evaluated as a single agent and in combination with letrozole in a breast cancer cell line engineered to express aromatase. The combination of taselisib and letrozole decreased cellular viability and increased apoptosis relative to either single agent. Signaling cross-talk between the PI3K and ER pathways was associated with efficacy for the combination. In a secreted factor screen, multiple soluble factors, including members of the epidermal and fibroblast growth factor families, rendered breast cancer cells non-responsive to letrozole. It was discovered that many of these factors signal through the PI3K pathway and cells remained sensitive to taselisib in the presence of the soluble factors. We also found that letrozole resistant lines have elevated PI3K pathway signaling due to an increased level of p110α, but are still sensitive to taselisib. These data provide rationale for clinical evaluation of PI3K inhibitors to overcome resistance to endocrine therapies in ER+ breast cancer.

## INTRODUCTION

Breast cancer is the leading cause of nonsmoking cancer-related death in women and continues to be a major health concern [1]. Although a number of genetic and environmental factors contribute to the development of mammary epithelial neoplasia and malignancy, a well-established root of breast cancer is persistent exposure to endogenous or exogenous estrogen [2, 3]. Anti-estrogens and aromatase inhibitors have therefore been a foundation of breast cancer treatment [4, 5]. Tamoxifen has been the most frequently prescribed drug both as adjuvant therapy after surgery and for the treatment of advanced disease, prolonging both disease-free and overall survival. Aromatase inhibitors, such as letrozole, however, have been shown to have superior efficacy to tamoxifen in both early and advanced breast cancer with response rates of 30%–50% as first-line metastatic therapy [6]. However, despite these advances, endocrine therapy is limited by relapse or inevitable disease progression in the metastatic setting.

Increased signaling activity of the phosphoinositide-3 kinase (PI3K)/Akt pathway is a frequent element in most cancers [7]. Activation of the pathway occurs following activating point mutations in the *PIK3CA* gene (encoding the PI3Kα isoform), which occur across the entire gene, but most frequently in the kinase and helical domains [8-10]. Genetic deletion or loss of function mutations within the tumor suppressor PTEN, a phosphatase with opposing function to PI3K, also increases PI3K pathway signaling [11]. These aberrations lead to increased downstream signaling through kinases such as Akt and increased activity of the PI3K pathway has been proposed as a hallmark of resistance to cancer treatment [12].

Therapeutic targeting of the PI3K pathway with small molecule inhibitors may have clinical benefit, either as single agents in PI3K-addicted cancers or used more broadly in combination with other conventional or targeted therapies. Several inhibitors targeting the PI3K pathway have now entered clinical trials [13-15]. Here we describe preclinical data for the selective PI3K inhibitor taselisib, also called GDC-0032 [16]. Taselisib potently inhibits PI3K pathway signaling and combines well with letrozole in an aromatase expressing cell line. In models of acquired letrozole resistance, we found that PI3K pathway activity was elevated, but could be blocked by taselisib. Moreover, under these conditions of acquired letrozole resistance we found the cells to be equally sensitive to taselisib. Letrozole resistant cells were subsequently cultured with increasing concentrations of taselisib to derive a model of dual resistance to endocrine/PI3K therapies. Under these conditions, the cells remained equally sensitive to taselisib in combination with a CDK4/6 inhibitor or docetaxel. Taken together, we have developed a model to evaluate the use of PI3K and endocrine therapies in aromatase inhibitor sensitive and refractory ER+ breast cancer cells and demonstrate the activity of a novel inhibitor of PI3K in this indication.

## RESULTS

### Combination of taselisib with letrozole decreases viability of aromatase-expressing MCF7 cells

**Figure 1:**
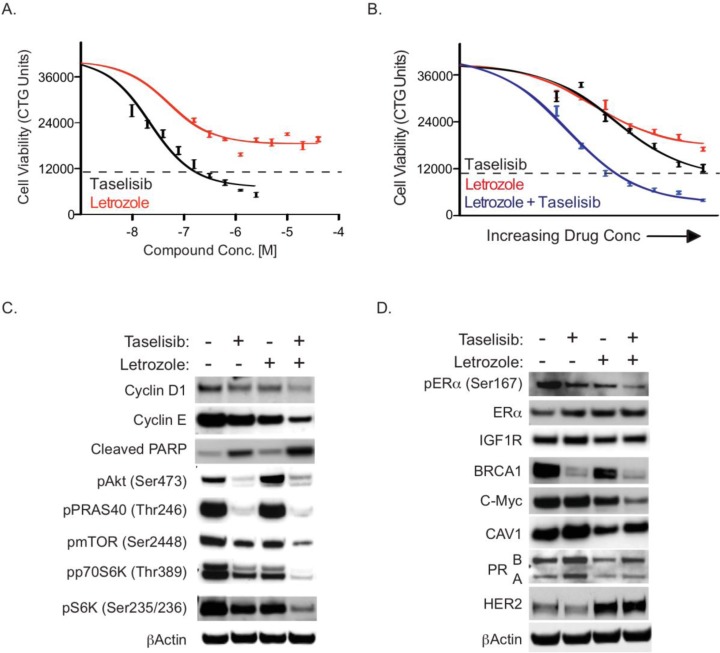
MCF7-ARO cells are sensitive to single agent and combination taselilsib and letrozole (A) Cell potency of taselisib and letrozole was determined in a 96-hour viability assay. (B) Taselisib combines well with letrozole in MCF7-ARO cells. The effect on viability of taselisib and letrozole as single agents is shown in the black and red lines, respectively. The combination effect of the two drugs is indicated with the blue line. (C) Increased growth arrest and apoptosis when taselisib and letrozole are combined. Immunoblots from MCF7-ARO samples treated for 24 hours with 0.4 μM taselisib and/or 0.6 μM letrozole. (D) Taselisib and letrozole independently influence the expression of well-known ER target genes. Treatments are for 0.4 hrs with 0.6 μM taselisib and/or 0.1μM letrozole. Dotted lines for all viability data are indicative of CellTiterGlo counts at the beginning of drug treatment. Error bars indicate standard deviation around the mean.

Taselisib, or GDC-0032, is a potent small-molecule inhibitor of class I PI3K isoforms, with reduced potency against the PI3Kβ isoform and with excellent selectivity against a large panel of other kinases including closely related family members DNA-PK, VPS34, c2α and c2β [16]. We sought to evaluate the combination effects of taselisib and letrozole in a preclinical breast cancer model expressing aromatase. MCF7 cells were transfected with an aromatase expression construct and put under neomycin drug selection to generate stable aromatase-expressing pools (MCF7-ARO). Significant levels of estrogen were detected in supernatants of stable pool cultures after the addition of androstenedione to the media (Supplementary Figure 1a). When grown in the presence of androstenedione, MCF7-ARO cells were more reliant on estrogen for growth as evidenced by increased sensitivity to all endocrine therapies evaluated (Figure 1A and Supplementary Figure 1B). MCF7-ARO cells were also quite sensitive to taselisib with an EC_50_ of 90 nM in viability assays (Figure 1A).

MCF7-ARO cells were treated with letrozole and taselisib in a dose titration starting at 4X EC_50_ single agent viability concentrations to determine if there is a combination effect between these two compounds. In comparison to single agent treatments, which approached EC_50_ levels at doses of 0.1 μM for letrozole (46% growth inhibition) and 0.1 μM taselisib (47% growth inhibition), the combination of taselisib and letrozole reduced MCF7-ARO viability by 81% (Figure 1B).

The effect of the compounds on downstream PI3K pathway markers was investigated with single agent and combination drug treatments at their EC_50_ concentrations at a 24-hour timepoint (Figure 1C). Taselisib caused a decrease of these pathway markers in the presence or absence of letrozole, as expected for an inhibitor of PI3K. We also detected an increase in phospho-Akt^Ser473^ in response to letrozole alone, which has been described previously for endocrine therapy treatment [17, 18]. Letrozole treatment modulated markers at mTOR and downstream which is consistent with previous reports of estrogen regulated processes [19].

To determine the effects of drug combinations, we looked at the cell cycle and apoptotic markers – cyclin D1, cyclin E, and cleaved poly (ADP-ribose) polymerase (PARP) after 24 hours of treatment (Figure 1C). Cyclin D1 and cyclin E are expressed in proliferating cells and help control the progression of cells through the cell cycle. Reduction of these cyclins was observed with single-agent treatments and was further decreased with combined presence of both agents. PARP is one of the main cleavage targets of caspase 3, and cleaved PARP serves as a marker of apoptotic cells [20]. A modest increase in this marker was detected with single agents, especially with taselisib treatment, but a substantial increase in cleaved PARP was detected with the combination.

To analyze the combination effects of taselisib and letrozole on ER signaling we first evaluated ERα phosphorylation on serine 167, a marker of ER activation that is downstream of PI3K signaling (Figure 1D) [21, 22]. MCF7-ARO cells were either vehicle-treated, treated with single agent taselisib or letrozole, or the combination of these agents for 24 hrs. We found that both inhibitors reduced phospho-ERα^Ser167^, and the combination was even more effective (Figure 1D). A small increase in total ER protein was also observed with letrozole and taselisib treatments. We also evaluated the expression of commonly described ER target genes with these treatments [23, 24, 25]. We observed a strong decrease in BRCA1 with PI3K pathway blockade that was consistent with previous reports in breast cancer models [26]. C-Myc protein, a marker downstream of both the PI3K and ER pathways was decreased with the two drugs in combination. We observed additional examples of cross-talk between the PI3K and ER pathways. Caveolin-1 (CAV1) and progesterone receptor (PGR) are two commonly described ER-regulated genes. Protein levels of these markers were decreased as expected with letrozole treatment, but these markers were increased with taselisib treatment. HER2 was increased with letrozole treatment, but not inhibition of PI3K.

### Multiple soluble factors that activate the PI3K pathway confer resistance to letrozole in MCF7-ARO cells

**Figure 2:**
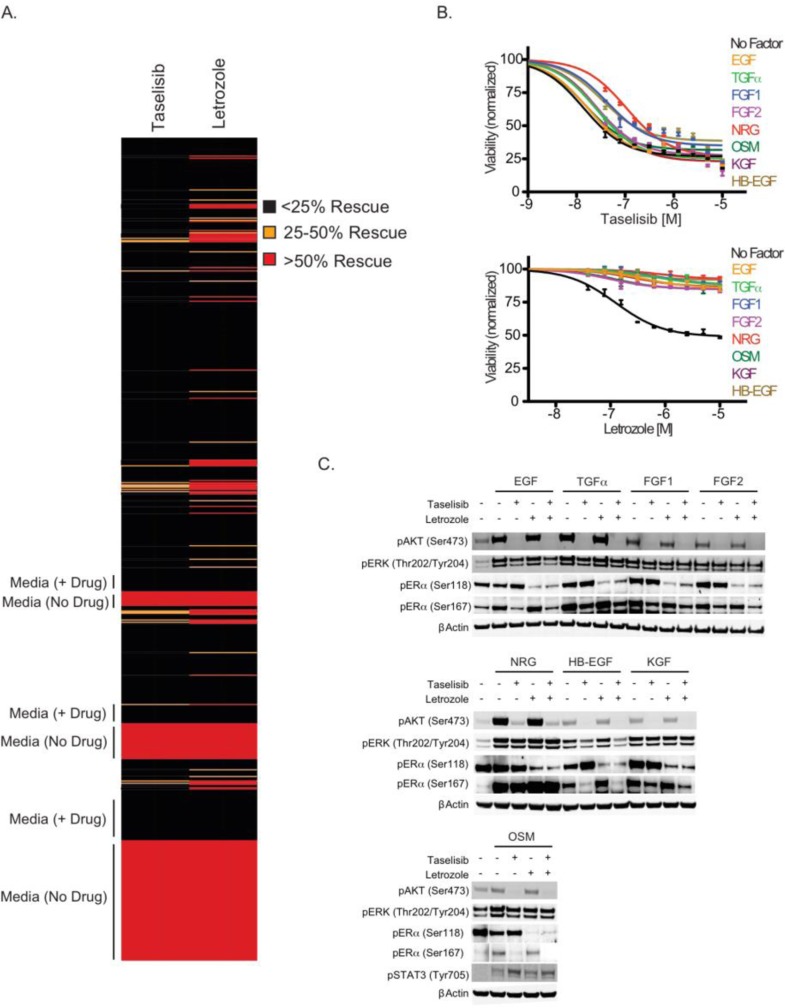
A subset of secreted factors mediate resistance to letrozole (A) MCF7-ARO cells treated with DMSO, single agent taselisib or letrozole plus media or 50 ng/ml of one of 418 secreted factors, and assayed for viability using Cell-TiterGlo. Controls in the absence of secreted factors in the presence or absence of taselisib are indicated. (B) Confirmation of factors that rescue growth inhibition by letrozole. MCF7-ARO cells treated with taselisib (top) or letrozole (bottom) plus 50 ng/ml of the indicated secreted factors, and assayed for viability using CellTiter-Glo. (C) Many of the secreted factors signal down the PI3K pathway. MCF7-ARO cells simulated with 50 ng/ml of the indicated secreted factors in the presence of DMSO, 0.6 μM taselisib, 1 μM letrozole or a combination of the two drugs. After 1 hr, cell lysates were prepared and analyzed by immunoblotting for pERK^T202/Y204^, pAKT^S473^, pERα^S118^, pERα^S167^,pSTAT3^Y705^, and βActin. For viability experiments, error bars indicate standard deviation around the mean.

We next investigated potential taselisib and letrozole resistance mechanisms due to factors secreted by the tumor microenvironment or other tissues. For these experiments we utilized a screen of commercially available factors to identify candidates that rescue taselisib-or letrozole-induced growth inhibition. For the screen, MCF7-ARO cells were dosed with a 1 μM concentration of taselisib or letrozole as well as 50 ng/ml of one of 418 soluble ligands for 72 hours (Supplementary Table 1). Four factors (FGF1, FGF2, KGF, and NRG) were able to rescue taselisib growth inhibition by greater than 25%. Factors that promoted resistance to letrozole were more common. We found that 26 factors (6.2% of total) were able to rescue letrozole-induced growth inhibition greater than 25% and 15 (3.6%) of those factors rescued growth inhibition above 50% (Figure 2A and Supplementary Table 1). To confirm the ability of these ligands to overcome letrozole-induced growth inhibition, eight factors were tested for their effect on the cellular potency of letrozole or taselisib in MCF7-ARO cells. Consistent with their activity in the large secreted factor screen, these factors promoted complete restoration of letrozole-induced growth inhibition (Figure 2B). Some of these factors also reduced the effectiveness of taselisib in viability experiments, but to a lesser degree. Inhibition was reduced approximately 2-fold by FGF1 and HB-EGF, while NRG resistance approached 3-fold.

To find the underlying mechanism of the ability of these ligands to decrease letrozole sensitivity, we investigated downstream signaling in the PI3K, MAPK and estrogen signaling pathways. All ligands increased the PI3K pathway marker pAKT^Ser473^ (Figure 2C). Letrozole treatment did not affect pAKT^Ser473^, but this marker was decreased in the presence of taselisib. Phospho-ERK1/2^T202/Y204^ was increased with secreted factor treatments in the presence or absence of taselisib or letrozole, suggesting the cells could use activation of the MAPK pathway under conditions of ligand stimulation. Estrogen receptor (ER) phosphorylation provides an important mechanism to regulate ER activity [21, 27]. Two well-described phosphorylation sites on ERα are serine 118 and 167, both located in the amino-terminal transcription activation domain. Increased phosphorylation of ERα on serine 167 was detected following stimulation with the evaluated soluble factors. Changes in pERα^Ser118^, however, were not detected with stimulation. Signaling changes elicited by inhibitor treatment converged on two key sites of post-translational modification of estrogen receptor. Phospho-ERα^Ser118^ was decreased with letrozole, while Phospho-ERα^Ser167^ was decreased with taselisib treatment.

Oncostatin M (OSM) is a member of the IL-6 family of cytokines and induces activation of the Jak2/Stat3 pathway [28]. Minor increases in pAKT^Ser473^ and pERα^Ser167^ were detected with OSM stimulation that could be inhibited by taselisib (Figure 2C). We also observed increased pSTAT3^Tyr705^ with OSM stimulation of MCF7-ARO cells that could not be blocked by taselisib or letrozole, which implies that activation of this alternative pathway may confer resistance to letrozole by this cytokine (Figure 2C).

### Letrozole resistant cells exhibit increased PI3K pathway signaling

**Figure 3:**
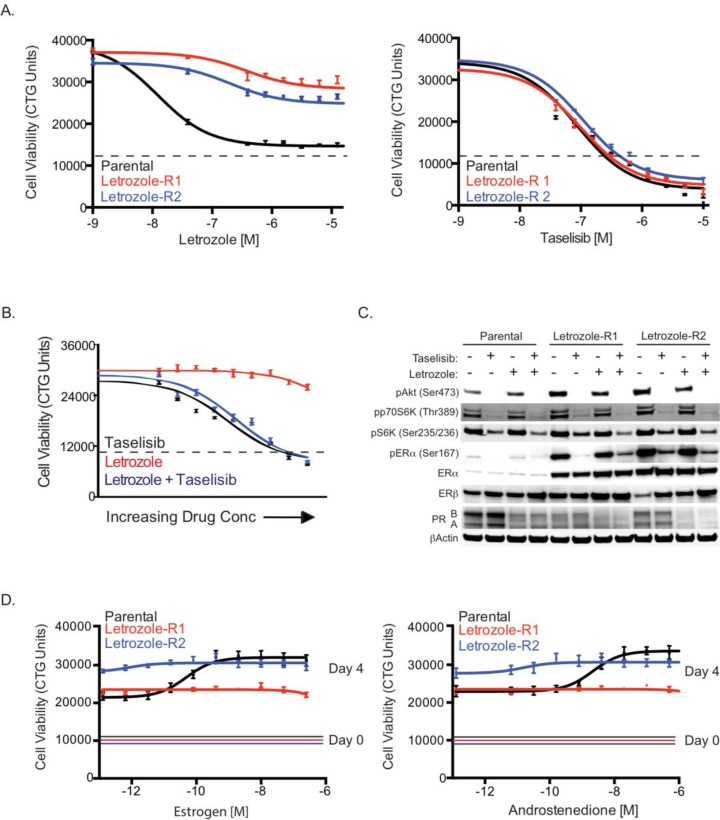
Characterization of letrozole resistant cells (A) MCF7-ARO parental or letrozole resistant cells treated with a dose titration of letrozole (left) or taselisib (right) and assayed for viability using CellTiter-Glo (CTG) 96 hrs post dosing. Dotted lines are indicative of CTG counts at the beginning of drug treatment. (B) Similar response to taselisib by itself or in combination with letrozole, in letrozole resistant cells. The effect on viability of taselisib and letrozole as single agents is shown in the black and red lines, respectively. The combination effect of the two drugs is indicated with the blue line. Dotted lines are indicative of CTG counts at the beginning of drug treatment. (C) Letrozole resistant cells have increased ERα. Treatments are for 24 hrs with 0.1 μM taselisib and/or 0.1μM letrozole. (D) Resistant cells do not increase proliferation in response to estrogen. MCF7-ARO parental or letrozole resistant cells cultured with a dose titration of androstenedione (right) or estrogen (left) and assayed for viability using CellTiter-Glo after 96 hrs. Solid lines are indicative of CTG counts prior to estrogen or androstenedione treatment. Error bars indicate standard deviation around the mean.

In addition to investigating the role of soluble ligands in acute or innate letrozole resistance, we sought to examine factors involved in acquired resistance to letrozole. MCF7-ARO cells were treated at increasing doses of letrozole over a period of 4 months and two resistant pools were generated. At the end of the dose escalation, the cells were able to grow at a letrozole concentration approximately 10-fold higher (6.25 μM) than the dose required for 50% growth inhibition of MCF7-ARO parental cells (0.625 μM). The generated resistant pools were significantly more resistant to letrozole and other estrogen therapies, compared to the parental MCF7-ARO line (Figure 3A and Supplementary Figure 2). Taselisib sensitivity in the letrozole resistant lines, however, was similar to the parental line. In combination experiments, letrozole resistant cells were equally sensitive to single agent taselisib or letrozole in combination with taselisib (Figure 3B).

**Figure 4:**
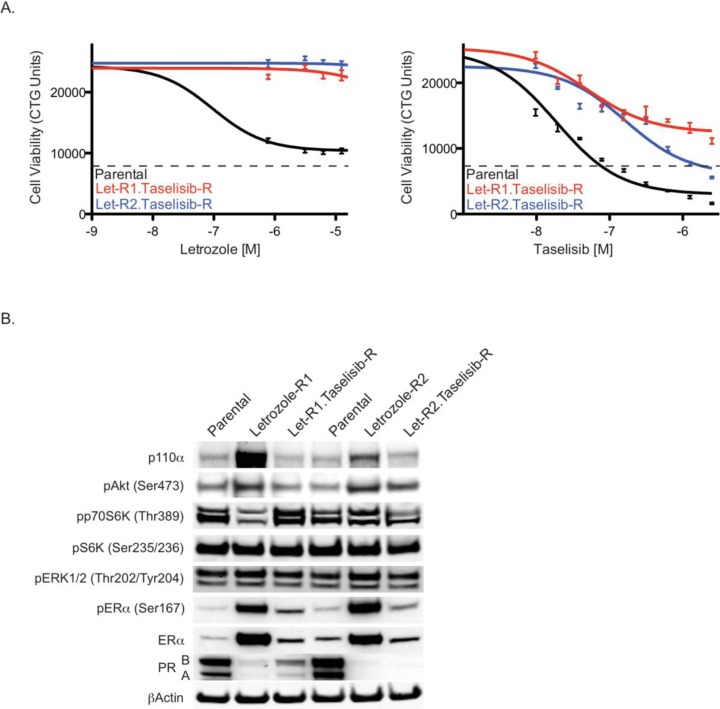
Characterization of cells resistant to both letrozole and taselisib (A) MCF7-ARO parental or taselisib/letrozole resistant cells treated with a dose titration of letrozole (left) or taselisib (right) and assayed for viability using CellTiter-Glo 96 hrs post dosing. (B) Protein changes with the acquisition of resistance to letrozole or dual resistance to letrozole and taselisib, compared to parental MCF7-ARO. Cell lysates were prepared and analyzed by immunoblotting for the markers indicated. Dotted lines for all viability data are indicative of CTG counts at the beginning of drug treatment. Error bars indicate standard deviation around the mean.

Once the resistant pools were confirmed to retain resistance to letrozole, signaling components of the PI3K and ER pathways were evaluated by western blot analysis. Both of the letrozole resistant pools were shown to have increased levels of the class I PI3K isoform p110α which was not associated with increased gene expression (Supplementary Figure 3 and Figure 4b). Not surprising, we observed augmented levels of phospho-Akt at the S473 phosphorylation site in the resistant pools, a marker proximal to the PI3K enzyme in the pathway. Other PI3K pathway components did not appear to increase, but all markers of this pathway were reduced with taselisib treatment (Figure 3C).

Changes in ERα protein levels have been described in long-term estrogen-deprived (LTED) models of endocrine resistance [29, 30]. In LTED models with increased ERα, an augmented response to estrogen stimulation was also observed for cell growth. In our letrozole resistant MCF7-ARO cells, we also detected significantly increased ERα, which correlated with increased phospho-ERα^Ser167^, but decreased PR (Figure 3C). We did not detect changes in ERβ in letrozole resistant MCF7-ARO cells. As expected, the elevated phospho-ERα^Ser167^ was diminished with taselisib treatment in the resistant cells. Interestingly, ERα levels did not correspond with a proliferative response to estrogen in our models. While the parental line exhibited a dose-response to added estrogen or androstenedione, letrozole resistant cells did not increase growth with either agent (Figure 3D).

We also investigated commonly described mechanisms of endocrine therapy resistance, such as MET and RET tyrosine kinases and FOSL1 transcriptional regulator [31, 32, 33]. Although we found the expression of some of these markers to be changed, using small molecule inhibitor and RNA interference knockdown studies we could not validate their involvement in resistance to endocrine therapies (Supplementary Figure 4).

Given that letrozole resistant MCF7-ARO models remained sensitive to taselisib, treatment of endocrine resistant tumors with taselisib may be appropriate and offer clinical benefit. Hence, we next wanted to model the possibility of eventual progression following the treatment of letrozole resistant cells with taselisib. Letrozole-R1 and –R2 cells were treated at increasing doses of taselisib over a period of 8 months. At the end of the dose escalation, the cells were able to grow in the presence of taselisib at a concentration greater than 25-fold higher (2.5 μM) than the initial EC_50_ dose (0.09 μM). The newly generated taselisib-resistant pools also remained highly resistant to letrozole, compared to the parental MCF7-ARO line (Figure 4A).

Once the pools were confirmed to be dually resistant to letrozole and taselisib, signaling components of the PI3K and ER pathways were evaluated by western blot analysis in comparison to parental and letrozole resistant pools. The elevated levels of pAkt^S473^, ERα and pERα^S167^ that were observed in letrozole resistant cells were reduced in the dually resistant cells to levels comparable to those of MCF7-ARO parental cells (Figure 4B). The letrozole resistant and dual resistant pools both had reduced PR compared to the parental cells.

### Cells resistant to both letrozole and taselisib remain sensitive to taselisib in combination with docetaxel or CDK4/6 inhibition

**Figure 5:**
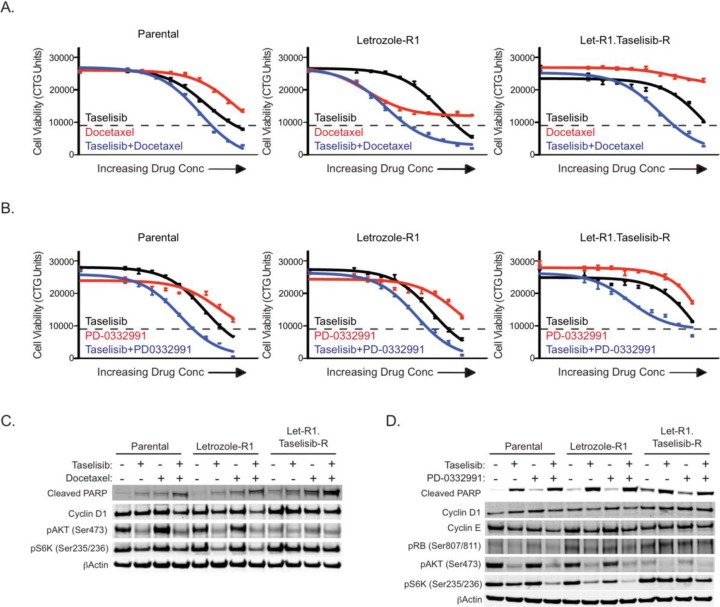
Dual resistant cells are still sensitive to taselisib in combination with docetaxel or CDK4/6 inhibition (A) Taselisib combines well with docetaxel in MCF7-ARO cells. The effect on viability of taselisib and docetaxel as single agents is shown in the black and red lines, respectively. The combination effect of the two drugs is indicated with the blue line. Starting doses for taselisib were 80 nM for the parental and letrozole-R1 lines and 10 μM for taselisib for Let-R1.GDC-0032-R. Docetaxel starting doses were 36 nM for all three lines. (B) Taselisib combines well with PD-0332991 in MCF7-ARO cells. The effect on viability of taselisib and PD-0332991 as single agents is shown in the black and red lines, respectively. The combination effect of the two drugs is indicated with the blue line. Starting doses for taselisib were 80 nM for the parental and letrozole-R1 lines and 10 μM for dual-resistant. PD-0332991 starting doses were 10 μM for all three lines. (C) Increased apoptosis is observed with the taselisib and docetaxel combination in cells sensitive or resistant to taselisib and letrozole. Immunoblots from samples treated for 24 hours with 20 nM taselisib (Parental and Letrozole-R1) or 2.5 μM (Let-R1.Taselisib-R) and/or 9 nM docetaxel. (D) Increased growth arrest is observed with combined PI3K and CDK4/6 inhibition. Immunoblots from samples treated for 24 hours with 20 nM taselisib (Parental and Letrozole-R1) or 2.5 μM (Let-R1.Taselisib-R) and/or 2.5 μM PD-0332991. Dotted lines for all viability data are indicative of CTG counts at the beginning of drug treatment. Error bars indicate standard deviation around the mean.

We sought to evaluate if letrozole resistant and dual letrozole/taselisib resistant cells were sensitive to other inhibitors that are being used to treat ER positive breast cancer patients [34]. Overall, we found that the parental and letrozole resistant cells remained sensitive to docetaxel and the CDK4/6 inhibitor PD-0332991, but these agents had reduced potency in dual resistant clones (Figure 5A and 5B).

To evaluate docetaxel in combination with taselisib, the MCF7-ARO parental, letrozole-R1 and Let-R1. GDC-0032-R dual resistant cells were treated with these agents in a dose titration for each line (Figure 5A). In these experiments, drug concentrations were the same across the cell lines. Decreased viability was observed relative to single agent treatments in each cell line model. Interestingly, docetaxel did not have an effect in the dual resistant cells as a single agent, but was effective in combination with taselisib.

The effect of the compounds on downstream PI3K pathway markers Akt and S6, the apoptotic marker cleaved PARP, and cyclin D1 were investigated with single agent and combination drug treatments at EC_50_ concentrations for the parental cell line at a 24-hour timepoint (Figure 5C). In the drug combination increased cleaved PARP and a small reduction in cyclin D1 was observed in the three cell line models. As expected, decreases in PI3K pathway signaling were observed with taselisib treatments.

We also evaluated PD-0332991 in combination with taselisib in parental and resistant clones (Figure 5B). PD-0332991 (Palbociclib) is a CDK4/6 small molecule inhibitor currently under evaluation in the clinic for ER+ breast cancer in combination with letrozole [34]. Relative to single agent treatments, decreased viability was observed with the PD-0332991 and taselisib combination in each cell line model similar to findings in other studies [35].

To determine the effects of the PD-0332991 and taselisib drug combination in parental and resistant cell line models, we assessed cyclin D1, cyclin E, phosphorylated Rb (Ser807/811) and cleaved PARP after 24 hours of treatment (Figure 5D). Cleaved PARP was detected with all taselisib treatments and a decrease in cyclin E was detected with the drug combination. Hyperphosphorylation of Rb at multiple sites, including 807 and 811 is indicative of cells that have entered the cell cycle and are proliferating. Both letrozole and dual letrozole/taselisib resistant cells had increased phosphorylation of Rb^Ser807/811^ that was decreased with PD-0332991 and taselisib combination drug treatment. This molecular mechanism is consistent with a recent report using additional PI3K and CDK4/6 inhibitors with MCF7 and T47D parental cells [35]. As expected, decreases in PI3K pathway signaling were observed with taselisib treatments.

## DISCUSSION

Breast cancer is a molecularly and clinically heterogeneous disease [36] and there remains a significant unmet medical need. Patients with ER-positive/HER2-negative tumors have clinical benefit when receiving hormone therapies, although recurrence can occur despite adjuvant endocrine treatment. Prolonged inhibition of ER signaling has been evaluated and several clinical trials have demonstrated the value of extended use of aromatase inhibitors, such as letrozole, following 5 years of tamoxifen treatment [37]. Another approach to address endocrine therapy resistance is to target the signaling crosstalk between ER and other pathways. Given that components of the PI3K pathway, and specifically the *PIK3CA* gene, are frequently mutated in breast cancer (77% and 35%, respectively) [38] and that crosstalk between ER and PI3K signaling pathways has been established [17, 39], we asked whether PI3K inhibition could decrease proliferation of breast cancer cells in the context of resistance to aromatase inhibition.

In our studies the PI3K inhibitor taselisib potently inhibited PI3K pathway signaling and combined well with letrozole in an aromatase-expressing breast cancer cell line (Figure 1). Our studies demonstrate that increased PI3K pathway activation confers resistance to letrozole. In a secreted factor screen, multiple soluble factors lead to letrozole resistance and these factors also increase PI3K signaling (Figure 2). PI3K pathway activity was also elevated in cells that were selected for acquired letrozole resistance, but the increased signaling could be reduced by taselisib treatment (Figure 3A). Moreover, under these conditions of acquired letrozole resistance the tumor cells were equally sensitive to taselisib. Letrozole-resistant cells expressed higher p110α protein levels, which may be responsible for the increase in PI3K pathway signaling (Figure 4B). Importantly, although letrozole resistant cells had increased expression levels of ER, these tumor lines did not utilize estrogen for growth and had decreased amounts of the ER target gene PGR, further suggesting that they were using alternative pathways for growth (Figure 4B).

**Figure 6:**
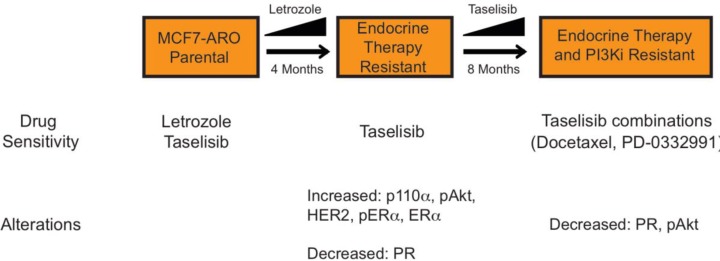
Underlying resistance mechanisms to letrozole and taselisib in the MCF-ARO model Drug sensitivity and cell alterations that accompany resistance are indicated.

The letrozole resistant cells were used to create a breast cancer cell line model that was resistant to both taselisib and letrozole (Figure 4). Interestingly, the elevated p110α and ER proteins observed in the letrozole resistant cells had returned to parental levels in the dual resistant cells. Despite resistance to letrozole or taselisib, the cells were sensitive to taselisib in combination with other ER+ breast cancer therapies, docetaxel and the PD-0332991 CDK4/6 inhibitor. Taken together, these data provide further rationale for evaluating PI3K pathway inhibitors for HR+ breast cancer treatment in the clinic (Figure 6).

Everolimus, an inhibitor of mTOR (which is a critical component of the PI3K pathway), has been shown to prolong progression-free survival in combination with the aromatase inhibitor, exemestane [40]. Encouraged by the efficacy and safety detected with concomitant inhibition of mTOR and ER signaling, a number of novel agents that target the PI3K pathway are currently in clinical trials, including the novel PI3K inhibitor, taselisib. In these cell line models of letrozole resistance, taselisib treatment alone or in combination with other therapies was able to re-sensitize breast cancer resistant models.

## MATERIALS AND METHODS

### Cell culture

MCF7 cell line was obtained from the American Type Culture Collection (ATCC, VA). The cells were tested and authenticated using gene expression and single nucleotide polymorphism genotyping arrays, as previously described [41, 42] and cultured in RPMI supplemented with 10% fetal bovine serum, 100 units/ml penicillin, 100 μg/ml streptomycin, 2mM L-glutamine and NEAA at 37°C under 5% CO. Stable aromatase-expressing MCF7 cells (MCF7-ARO) were generated by transfection of a plasmid vector containing the full aromatase gene and a neomycin selection gene. The cells were maintained in androstenedione and all experiments were performed in the presence of androstenedione except where indicated (Figure 3D).

### Materials

Taselisib, also called GDC-0032, was generated at Genentech, Inc. (South San Francisco, CA). Letrozole was obtained from US Biological. Antibodies used include phospho-AKT^Ser473^, AKT, phospho-PRAS40^Thr246^, phospho-S6^Ser235/236^, phospho-S6^Ser240/242^, S6, phospho-ERK^Thr202/Tyr204^, ERK, phospho-ERα^Ser118^, phospho-ERα^Ser167^, cleaved PARP, p110α, phospho-p70S6K^Thr389^, PR, cyclin E, phospho-mTOR^Ser2448^, IGF1R, BRCA1, c-Myc, CAV1, HER2 and cyclin D1 obtained from Cell Signaling (Danvers, MA). Antibodies for ERα and ERβ were obtained from Santa Cruz biotechnology (Santa Cruz, CA) and a βActin antibody was obtained from Sigma (St. Louis, MO).

### Cell viability assays

384-well plates were seeded with 2000 cells/well in a volume of 54 μl per well followed by incubation at 37°C under 5% CO overnight (∼16 hours). Compounds were diluted in DMSO to generate the desired stock concentrations then added in a volume of 6 μL per well. All treatments were tested in quadruplicate. After 4 days incubation, relative numbers of viable cells were estimated using CellTiter-Glo (Promega, Madison, WI) and total luminescence was measured on an Envision plate Reader (PerkinElmer, Foster City, CA). The concentration of drug resulting in 50% inhibition of cell viability (IC_50_) or 50% maximal effective concentration (EC_50_) was determined using Prism software (GraphPad, La Jolla, CA). For cell lines that failed to achieve an EC_50_ the highest concentration tested (10 μM) is listed.

### Letrozole resistant cell line selection

MCF7-ARO cells were grown in increasing concentrations of letrozole in the presence of androstenedione in phenol red free RPMI medium, supplement with 10% Charcoal dextran stripped FBS, until they grew normally in a letrozole concentration of 6.5 μmol/L. For cells resistant to both letrozole and taselisib, letrozole resistant cells were grown in increasing concentrations of the taselisib, until they grew normally in a concentration of 2.5 μmol/L. Maintenance of aromatase expression in all letrozole sensitive and resistant clones was verified using TaqMan.

### Protein assays

10 cm^2^ dishes were seeded with two million cells in a volume of 10 mL followed by incubation at 37°C under 5% CO_2_ overnight (∼16 hours). Cells were treated with the indicated concentration of taselisib or pictilisib for the time indicated. Following treatment, cells were washed with cold PBS and lysed in 1X Cell Extraction Buffer from Invitrogen (Carlsbad, CA) supplemented with protease inhibitors (Roche, Germany), Phosphatase Inhibitor Cocktails 2 and 3 from Sigma (St. Louis, MO). Protein concentration was determined using the Pierce BCA Protein Assay Kit (Rockford, IL). For immunoblots, equal protein amounts were separated by electrophoresis through NuPage Bis-Tris 4-12% gradient gels (Invitrogen, Carlsbad, CA); proteins were transferred onto nitrocellulose membranes using the iBlot system from Invitrogen (Carlsbad, CA).

### Secreted factor screen

Recombinant purified secreted factors were purchased from Peprotech and R&D Systems as indicated, and were reconstituted in PBS/0.1% BSA (Supplementary Table 1). Secreted factors were transferred into 96-well plates at a concentration of 1 μg/ml, and subsequently diluted to 100 ng/ml in media containing either no drug or 0.6 μM of taselisib or 1 μM letrozole. Equal volumes of diluted factor (final concentration 50 ng/ml) were arrayed into the 384 well plates pre-seeded with cells (2000 cells per wells seeded the day before) using a Bravo liquid handler. After 72 hours incubation, cell viability was determined using CellTiter-Glo (Promega).

### Statistics

Significant differences comparing lines with and without evaluated genetic abnormalities was determined by two-tailed Mann-Whitney test calculated using the JMP statistical software, version 5.1.2 and p values reported (JMP Software, Cary, NC).

## CONCLUSIONS

The results of this study suggest that selective PI3K inhibition, either alone or in combination with other breast cancer treatment modalities, may be efficacious in HR+ tumors that are either sensitive or refractory to single agent endocrine therapy such as letrozole treatment.

## SUPPLEMENTARY FIGURES AND TABLE



## Abbreviations

PI3K: phosphoinositide-3 kinase
HR: hormone receptor
ER: estrogen receptor
PR: progesterone receptor
PGR: progesterone receptor gene
CDK: cyclin dependent kinase
EC: effective concentration
IC: inhibitory concentration
OSM: oncostatin M
LTED: long term estrogen deprived
