# Supplementary material for: The PI3K inhibitor taselisib overcomes letrozole resistance in a breast cancer model expressing aromatase
Source: Genes Cancer. 2016 Mar;7(3-4):73–85. doi: 10.18632/genesandcancer.100 (PMC4918946; doi:10.18632/genesandcancer.100)
Supplement: Supplementary file 1 [file ganc-07-073-s001.pdf]

The PI3K inhibitor taselisib overcomes letrozole resistance in a breast cancer model expressing aromatase

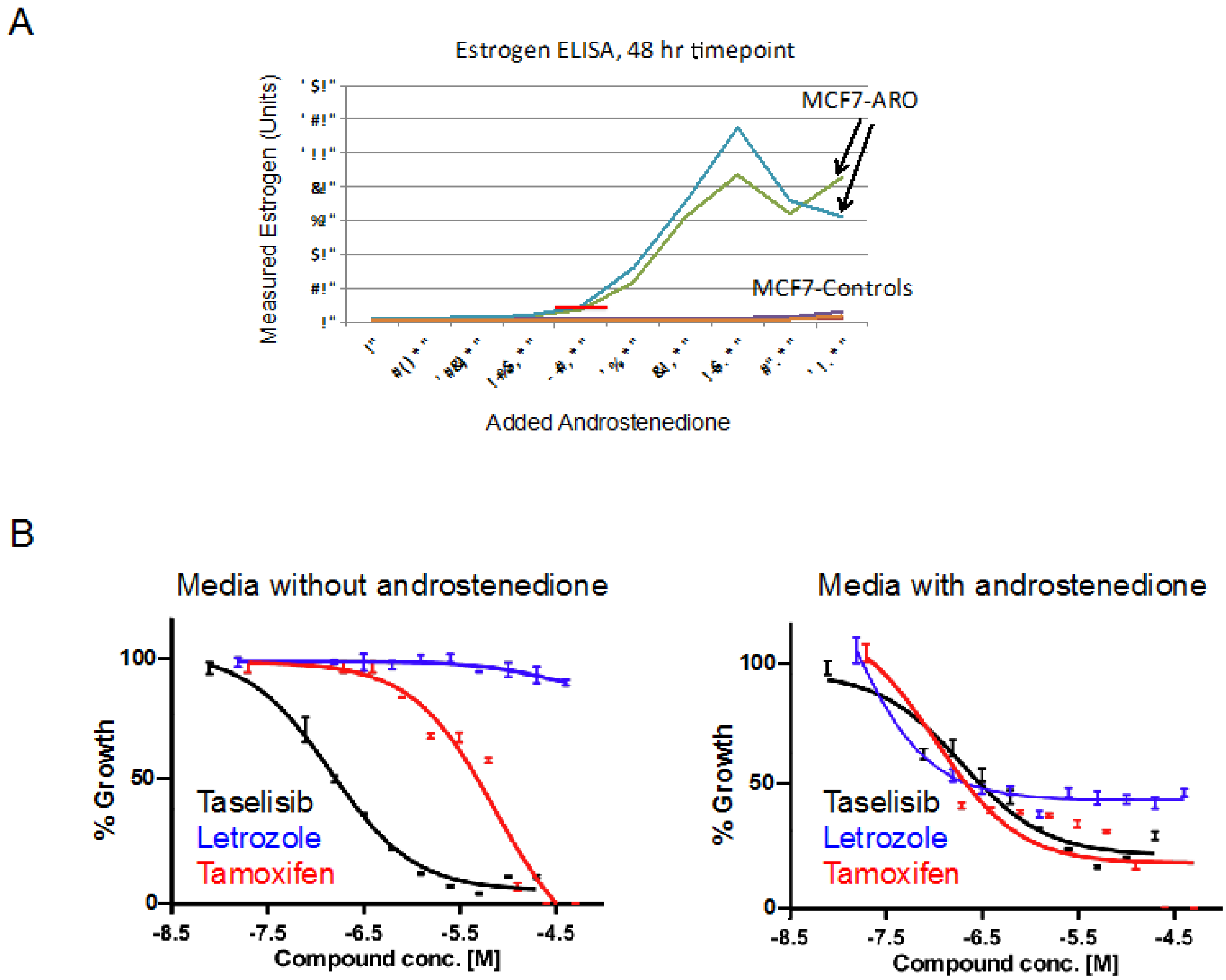

Supplementary Figure 1: MCF7 cells that stably express aromatase produce estrogen in response to androstenedione and become sensitive to inhibitors of the estrogen signaling pathway. (A) Estrogen production as measured by ELISA from MCF7 parental controls and MCF7-ARO cells. (B) Cell potency of taselisib, letrozole, and tamoxifen was determined in a 96-hour viability assay. Error bars indicate standard deviation around the mean.

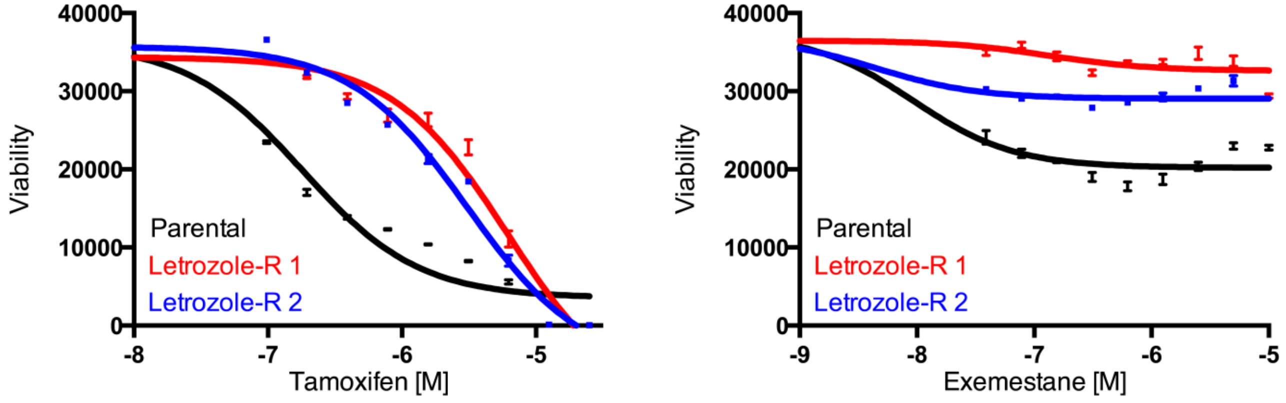

Supplementary Figure 2: MCF7-ARO Resistant pools are significantly more resistant estrogen therapies, compared to the parental MCF7-ARO line. Cell potency of tamoxifen and exemestane was determined in a 96-hour viability assay. Error bars indicate standard deviation around the mean.

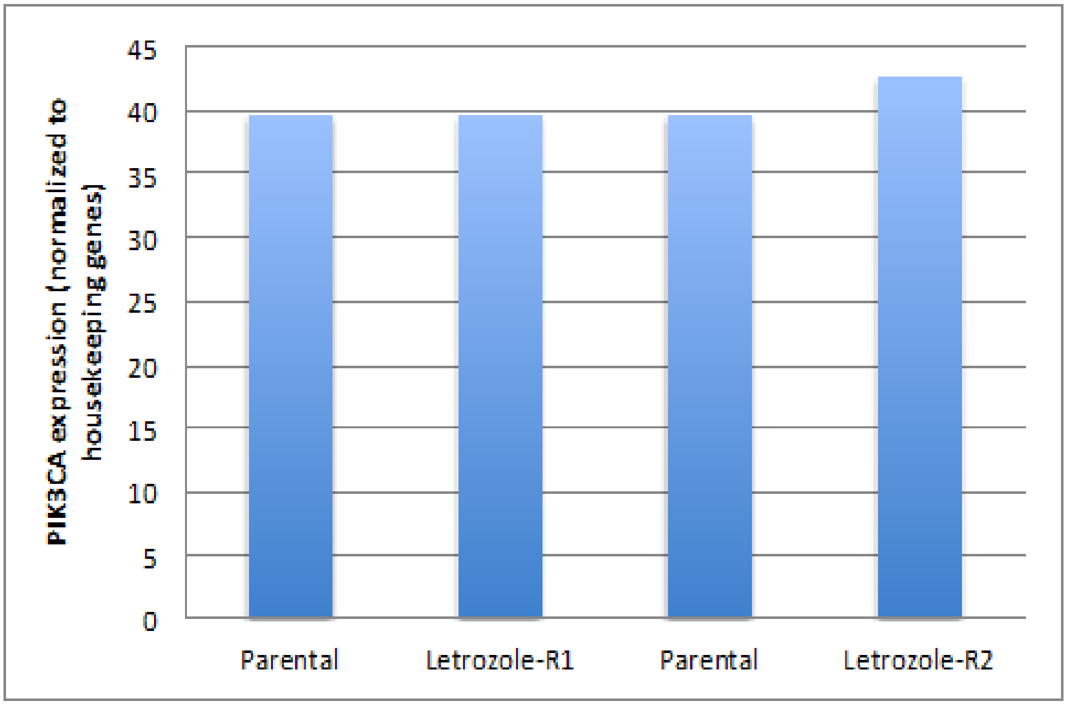

Supplementary Figure 3: Increased p110α protein expression is not due to increased gene expression. PIK3CA expression (normalized to housekeeping genes) was determined in parental and letrozole resistant MCF7-ARO clones.

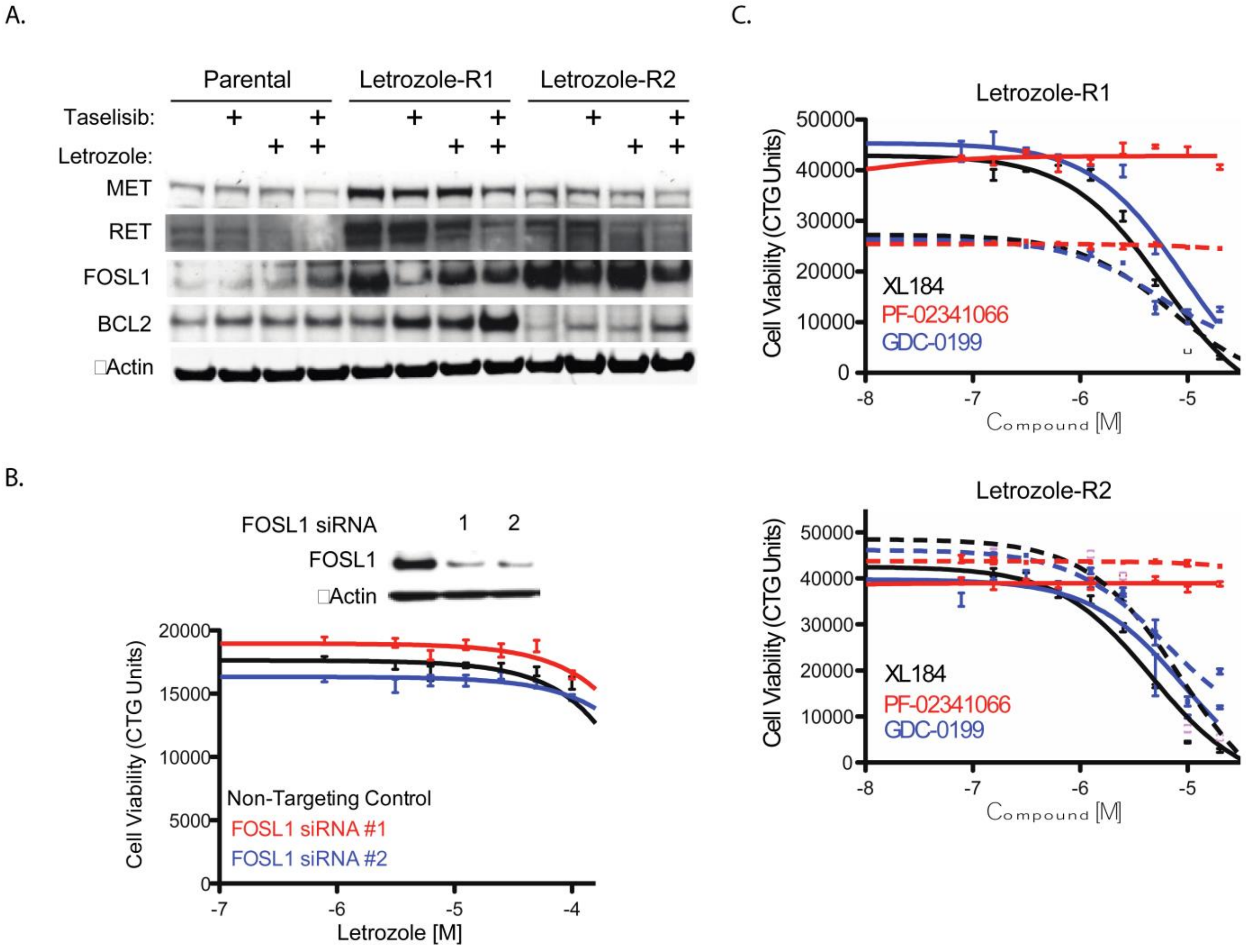

Supplementary Figure 4: Commonly described mechanisms of endocrine therapy resistance do suggest mechanisms of resistance in the MCF7-ARO line. (A) Increasses in MET, RET, FOSL1, and BCL2 are detected in letrozole resistant MCF7-ARO cells. Protein changes with letrozole and taselisib treatments are shown by immunoblot. Treatments are for 24 hrs with 0.1 μM taselisib and/or 0.1μM letrozole. (B) siRNA Knockdown of FOSL1 does not sensitize. Immunoblots for FOSL1 are from the 48 hr timepoint. Cell potency of letrozole in the MCF7-ARO letrozole resistant cell line was determined in a 96-hour viability assay. (C) Sensitivity of XL184, PF-02341066, and GDC-0199 in the letrozole resistant MCF7-ARO cells. Cell potency of compounds was determined in a 96-hour viability assay.

Supplementary Table 1: Secreted Factor Screen Results. Percent changes in viability of drug treated samples compared to drug treated samples in the presence of a secreted factor. Each row is for a single secreted factor.

	GDC-0032 (1	GDC-0032 (1 μM)	Letrozole (1 μM)	Letrozole (1 μM)
**4-1BB Ligand**	7%	-3%	-4%	-8%
**4-1BB Receptor**	7%	2%	14%	19%
**Activin-A (Insect cell derived)**	4%	-10%	-54%	-50%
**Activin-B (Insect cell derived)**	2%	-9%	-29%	-29%
**AITRL**	8%	0%	-6%	-24%
**Amphiregulin (98 a.a.)**	-3%	4%	16%	5%
**ANG-1 (HeLa cell derived)**	5%	0%	-5%	-16%
**ANG-2 (CHO cell derived)**	6%	-2%	-14%	-2%
**APRIL (insect cell derived)**	10%	1%	-16%	-24%
**Artemin**	5%	-4%	-12%	-11%
**BAFF**	7%	-3%	-12%	-3%
**BAFF Receptor**	-3%	0%	1%	-6%
**BCA-1/BLC (CXCL13)**	5%	-2%	-25%	-21%
**BCMA**	5%	-4%	-10%	-19%
**BDNF**	7%	-3%	-24%	-15%
**beta-NGF**	5%	-1%	17%	20%
**Betacellulin**	19%	13%	81%	86%
**BMP-1/PCP**	-18%	-18%	-28%	-15%
**BMP-13/CDMP-2**	5%	12%	56%	65%
**BMP-2**	6%	-4%	-32%	-36%
**BMP-3**	-4%	0%	2%	5%
**BMP-4 (HeLa cell derived)**	1%	-15%	-46%	-38%
**MP-4 (truncated; E.coli derive**	1%	-7%	-37%	-30%
**BMP-6 (HEK 293 cell derived)**	3%	-7%	-51%	-40%
**BMP-7 (CHO cell derived)**	8%	-3%	-14%	-9%
**BMP-8a**	-13%	0%	12%	-7%
**BMP-9**	-16%	-12%	-38%	-40%
**BRAK (CXCL14)**	4%	0%	6%	-11%
**Cardiotrophin-1**	15%	5%	8%	17%
**CCL1/I-309/TCA-3**	-18%	-17%	5%	6%
**CCL14a/HCC-1**	-9%	-3%	13%	-5%
**CCL18/PARC**	-8%	2%	28%	17%
**CCL2/JE/MCP-1**	-3%	4%	8%	15%
**CCL3/MIP-1 alpha**	-4%	-1%	-5%	5%
**CCL4/MIP-1 beta**	-3%	1%	9%	9%
**CCL5/RANTES**	-5%	3%	12%	5%
**CCL7/MCP-3/MARC**	-9%	-1%	4%	-5%

CCL8/MCP-2	-7%	2%	-6%	13%
CD22 (CHO cell derived)	7%	2%	-5%	-4%
CDNF	-4%	-5%	-11%	-18%
Chemerin	1%	-3%	-3%	-14%
CNTF	4%	-5%	-15%	-11%
CTACK (CXL27)	5%	-2%	-7%	-10%
CTGF	5%	-2%	-14%	-7%
CTGFL/WISP-2	3%	-2%	12%	-4%
CXCL1/GRO alpha/KC/CINC-1	-6%	1%	23%	20%
CXCL10/IP-10/CRG-2	-4%	5%	51%	26%
CXCL12/SDF-1 alpha	-6%	4%	30%	-3%
CXCL12/SDF-1 beta	-3%	6%	7%	3%
CXCL16	0%	-5%	-5%	-8%
:XCL2/GRO beta/MIP-2/CINC-	-5%	-2%	3%	1%
CL3/GRO gamma/CINC-2/DCII	-4%	1%	-6%	1%
CXCL7/NAP-2	-7%	5%	0%	-2%
CXCL8/IL-8	-3%	1%	6%	11%
CXCL8/IL-8	-11%	-3%	16%	6%
CYR61	10%	4%	30%	26%
DKK-1 (HEK293 cells)	8%	0%	-16%	-35%
E-Selectin (CHO cell derived)	0%	-1%	12%	29%
EG-VEGF	6%	1%	-4%	-9%
EGF (CON)	23%	8%	106%	102%
EGF (CON)	13%	13%	111%	109%
EGF (CON)	23%	11%	100%	114%
EGF (CON)	10%	8%	128%	121%
GF Receptor (CHO cell derivec	11%	1%	-12%	-23%
ENA-78/CXCL5 (5-78 a.a.)	6%	-2%	-22%	2%
ENA-78/CXCL5 (8-78 a.a.)	3%	-5%	-10%	-18%
Endostatin	-4%	2%	-5%	-6%
Eotaxin (CCL11)	7%	-2%	-15%	-10%
Eotaxin-2 (CCL24)	7%	-3%	-10%	0%
Eotaxin-3 (CCL26)	4%	-3%	-20%	-5%
Epigen	0%	-4%	-8%	-10%
Epiregulin	0%	1%	50%	46%
Exodus-2 (CCL21)	7%	-5%	4%	3%
ıin A/AHSG (HEK293 cell deriv	-1%	1%	19%	41%
FGF-10	11%	21%	133%	136%
FGF-16	-3%	6%	2%	-2%
FGF-17	-3%	0%	10%	6%
FGF-18	-2%	-1%	8%	38%

FGF-19	-6%	-2%	-10%	-3%
FGF-20	8%	-2%	-4%	0%
FGF-21	-6%	-2%	-3%	-10%
FGF-23	6%	3%	28%	16%
FGF-4	9%	6%	50%	74%
FGF-5	-4%	-3%	1%	1%
FGF-6	-3%	-2%	42%	33%
FGF-8	5%	12%	65%	86%
FGF-9	15%	7%	83%	68%
FGF-acidic	9%	23%	116%	124%
FGF-basic	19%	27%	124%	124%
FGF-basic (CON)	36%	21%	93%	118%
FGF-basic (CON)	22%	23%	112%	132%
FGF-basic (CON)	34%	20%	118%	131%
FGF-basic (CON)	26%	26%	142%	141%
Flt-3 Ligand	-7%	-7%	-18%	7%
Flt3-Ligand	9%	3%	-24%	-22%
Follistatin	5%	-1%	10%	9%
Fractalkine (CX3CL1)	2%	-8%	-20%	-9%
G-CSF	5%	-4%	-30%	-33%
gAcrp30/Adipolean	-5%	8%	6%	2%
gAcrp30/Adipolean Variant	-6%	1%	4%	7%
Galectin-1	-9%	-3%	-12%	4%
Galectin-3	4%	7%	18%	51%
GCP-2 (CXCL6)	2%	-5%	-16%	-18%
GDF-11	0%	-9%	-12%	-15%
F-15/MIC-1 (cell culture deriv	7%	-6%	-16%	-1%
GDF-2 (CHO cell derived)	-3%	-18%	-60%	-57%
GDF-3	5%	-7%	4%	-3%
GDF-5 (BMP-14/CDMP-1)	-6%	-6%	0%	-6%
GDF-7	6%	-2%	-37%	-38%
GDNF	12%	1%	14%	15%
GM-CSF	6%	3%	24%	22%
GMF-beta	11%	3%	7%	11%
GRO-beta (CXCL2)	11%	8%	6%	1%
GRO-gamma (CXCL3)	7%	-7%	-8%	4%
GRO/MGSA (CXCL1)	-6%	-3%	-13%	-12%
HB-EGF	12%	14%	125%	145%
HCC-1/CCL14 (66 a.a.)	10%	-2%	-14%	-17%
HCC-1/CCL14 (72 a.a.)	4%	-4%	-24%	-28%
Heregulin-beta1	31%	37%	165%	192%

HGF (CON)	18%	0%	11%	2%
HGF (CON)	7%	2%	23%	10%
HGF (CON)	16%	1%	27%	8%
HGF (CON)	2%	3%	19%	11%
HGF (Insect cell derived)	9%	-6%	-9%	-22%
HVEM-Fc (Insect cell derived)	-17%	-16%	-23%	-28%
I-309 (CCL1)	10%	0%	-36%	-30%
I-TAC (CXCL11)	5%	3%	17%	3%
ICAM-1 (CHO cell derived)	2%	7%	39%	38%
IFN-beta (CHO cell derived)	-18%	-27%	-48%	-53%
IFN-gamma	-28%	-27%	-65%	-46%
IFN-lambda 1	0%	0%	-3%	-6%
IFN-lambda 2	2%	-2%	0%	-9%
IFN-omega	-16%	-12%	-35%	-26%
IGF-BP1	-4%	-1%	-11%	-1%
IGF-BP2 (insect cell derived)	5%	-1%	-1%	-11%
IGF-BP3	-3%	0%	-8%	-7%
IGF-BP4 (insect cell derived)	3%	-4%	-5%	-6%
IGF-BP5	-5%	-7%	-11%	-3%
IGF-BP6 (insect cell derived)	4%	-3%	-3%	-13%
IGF-BP7	-1%	1%	12%	19%
IGF-I	-7%	-6%	6%	5%
IGF-II	-3%	3%	55%	76%
IL-1 beta/IL-1F2	-21%	-21%	-10%	16%
IL-10	7%	-4%	-13%	-5%
IL-11	11%	6%	-2%	9%
IL-12	0%	6%	78%	51%
IL-12p70 (CHO cell derived)	7%	-2%	-24%	-31%
IL-12p80 (insect cell derived)	9%	0%	-18%	-13%
IL-13	4%	-6%	-31%	-40%
IL-13 Variant	6%	-3%	-30%	-25%
IL-15	9%	-1%	-29%	-12%
IL-16 (121 a.a.)	5%	-5%	-36%	-32%
IL-16 (130 a.a.)	7%	-4%	-10%	-8%
IL-17A	-1%	6%	58%	46%
IL-17B	-1%	1%	11%	-1%
IL-17D	-1%	0%	8%	-8%
IL-17E	-3%	-2%	6%	-12%
IL-17F	-9%	-3%	2%	17%
IL-19	12%	9%	8%	10%
IL-1alpha	-16%	-17%	16%	-4%

**IL-1beta**	-15%	-20%	4%	-1%
**IL-1RA**	-8%	-1%	-14%	0%
**IL-2**	-5%	3%	4%	-6%
**IL-20**	3%	-4%	-18%	-5%
**IL-21**	5%	-1%	-10%	2%
**IL-22**	4%	-5%	-21%	-14%
**IL-23 (insect cell derived)**	4%	0%	-31%	-17%
**IL-3**	8%	-7%	-6%	1%
**IL-31**	7%	4%	10%	-2%
**IL-33**	-13%	-16%	7%	18%
**IL-34 (HEK293 cells)**	-7%	-11%	5%	-11%
**IL-4**	1%	1%	12%	-6%
**IL-5**	6%	-2%	-14%	-17%
**IL-6**	-14%	-15%	-15%	-44%
**IL-7**	-3%	4%	-5%	-29%
**IL-8 (72 a.a.) (CXCL8)**	-4%	3%	7%	4%
**IL-8 (77 a.a.) (CXCL8)**	-2%	-2%	1%	8%
**IL-9**	6%	-3%	-6%	-27%
**IP-10 (CXCL10)**	-3%	0%	16%	18%
**KGF (FGF-7)**	33%	26%	116%	133%
**KLOTHO (CHO cell derived)**	1%	1%	-4%	4%
**LAG-1 (CCL4L1)**	5%	0%	9%	17%
**LD78-beta (CCL3L1)**	1%	2%	13%	20%
**LEC/NCC-4 (CCL-16)**	3%	-2%	-4%	5%
**Leptin**	-10%	10%	3%	-10%
**LIGHT (Insect cell derived)**	5%	-2%	10%	11%
**Lymphotactin (XCL1)**	4%	-2%	4%	6%
**M-CSF**	9%	-4%	-21%	-22%
**MANF**	-3%	1%	19%	15%
**Maspin**	-1%	-4%	6%	3%
**MCP-1/MCAF (CCL2)**	0%	2%	18%	10%
**MCP-2 (CCL8)**	7%	1%	20%	9%
**MCP-3 (CCL7)**	4%	2%	26%	-1%
**MCP-4 (CCL13)**	6%	0%	3%	9%
**MDC (67 a.a.) (CCL22)**	2%	0%	-3%	14%
**MDC (69 a.a.) (CCL22)**	7%	-5%	9%	6%
**MEC (CCL28)**	4%	1%	13%	2%
**Media (+ DRUG)**	11%	6%	8%	0%
**Media (+ DRUG)**	4%	2%	10%	26%
**Media (+ DRUG)**	13%	4%	16%	23%
**Media (+ DRUG)**	-2%	3%	9%	7%

**Media (+ DRUG)**	6%	8%	5%	1%
**Media (+ DRUG)**	-10%	-15%	-2%	6%
**Media (+ DRUG)**	6%	0%	-3%	-11%
**Media (+ DRUG)**	8%	0%	-25%	-8%
**Media (+ DRUG)**	7%	-2%	-42%	-55%
**Media (+ DRUG)**	7%	-3%	-15%	-16%
**Media (+ DRUG)**	6%	-1%	-32%	-11%
**Media (+ DRUG)**	3%	-3%	-19%	-20%
**Media (+ DRUG)**	7%	5%	17%	26%
**Media (+ DRUG)**	1%	4%	19%	-11%
**Media (+ DRUG)**	1%	0%	-5%	-10%
**Media (+ DRUG)**	1%	-4%	-4%	-2%
**Media (+ DRUG)**	-2%	-3%	-11%	7%
**Media (+ DRUG)**	3%	-4%	-7%	-7%
**Media (+ DRUG)**	2%	-3%	-12%	-1%
**Media (+ DRUG)**	1%	-5%	-8%	12%
**Media (+ DRUG)**	-1%	-1%	23%	15%
**Media (+ DRUG)**	-1%	5%	7%	-8%
**Media (+ DRUG)**	-3%	-1%	4%	-5%
**Media (+ DRUG)**	-7%	0%	-14%	-10%
**Media (+ DRUG)**	-6%	2%	1%	0%
**Media (+ DRUG)**	-2%	-1%	-5%	0%
**Media (+ DRUG)**	-2%	-4%	-12%	-3%
**Media (+ DRUG)**	-2%	0%	0%	-15%
**Media (+ DRUG)**	-7%	4%	20%	16%
**Media (+ DRUG)**	-4%	5%	25%	-2%
**Media (+ DRUG)**	-8%	6%	3%	10%
**Media (+ DRUG)**	-7%	2%	7%	2%
**Media (+ DRUG)**	-3%	-6%	4%	17%
**Media (+ DRUG)**	-5%	0%	20%	2%
**Media (+ DRUG)**	-7%	-2%	-3%	7%
**Media (+ DRUG)**	-10%	3%	22%	17%
**Media (NO DRUG)**	109%	105%	93%	104%
**Media (NO DRUG)**	88%	117%	110%	111%
**Media (NO DRUG)**	111%	103%	100%	106%
**Media (NO DRUG)**	77%	104%	111%	112%
**Media (NO DRUG)**	115%	77%	110%	106%
**Media (NO DRUG)**	92%	100%	97%	102%
**Media (NO DRUG)**	112%	101%	103%	104%
**Media (NO DRUG)**	96%	103%	98%	103%
**Media (NO DRUG)**	107%	103%	107%	86%

**Media (NO DRUG)**	98%	106%	99%	86%
**Media (NO DRUG)**	114%	95%	87%	107%
**Media (NO DRUG)**	98%	100%	99%	92%
**Media (NO DRUG)**	111%	98%	106%	102%
**Media (NO DRUG)**	93%	100%	94%	93%
**Media (NO DRUG)**	109%	99%	107%	97%
**Media (NO DRUG)**	97%	93%	92%	97%
**Media (NO DRUG)**	114%	102%	98%	102%
**Media (NO DRUG)**	85%	103%	102%	111%
**Media (NO DRUG)**	106%	100%	85%	99%
**Media (NO DRUG)**	79%	96%	101%	92%
**Media (NO DRUG)**	107%	103%	95%	95%
**Media (NO DRUG)**	89%	98%	75%	99%
**Media (NO DRUG)**	106%	95%	94%	89%
**Media (NO DRUG)**	147%	158%	219%	223%
**Media (NO DRUG)**	100%	97%	94%	80%
**Media (NO DRUG)**	81%	87%	97%	90%
**Media (NO DRUG)**	102%	88%	93%	90%
**Media (NO DRUG)**	81%	95%	87%	85%
**Media (NO DRUG)**	104%	92%	76%	79%
**Media (NO DRUG)**	81%	99%	90%	85%
**Media (NO DRUG)**	107%	86%	87%	79%
**Media (NO DRUG)**	82%	95%	96%	93%
**MIA**	3%	-2%	11%	6%
**MIA-2**	6%	-6%	8%	2%
**Midkine**	3%	-4%	6%	11%
**MIG (CXCL9)**	1%	-1%	29%	8%
**MIP-1alpha (CCL3)**	6%	1%	26%	-9%
**MIP-1beta (CCL4)**	4%	1%	-15%	-7%
**MIP-3 (CCL23)**	4%	-2%	14%	-12%
**MIP-3alpha (CCL20)**	2%	0%	-2%	3%
**MIP-3beta (CCL19)**	5%	-3%	16%	0%
**MIP-4 (CCL18)**	7%	1%	42%	30%
**MIP-5 (CCL15)**	-4%	5%	0%	-1%
**Myostatin**	-1%	-2%	-2%	-10%
**Myostatin Propeptide**	-1%	2%	-12%	15%
**NAP-2 (CXCL7)**	8%	0%	-12%	-19%
**Neuritin**	7%	-4%	-2%	6%
**Neurturin**	13%	1%	18%	46%
**NNT-1/BCSF-3**	10%	-5%	-16%	-10%
**NOGGIN (293 cell derived)**	5%	1%	29%	3%

NOV	-1%	-3%	-3%	-2%
NRG1 beta 1 (CON)	34%	20%	131%	164%
NRG1 beta 1 (CON)	24%	19%	151%	194%
NRG1 beta 1 (CON)	33%	24%	147%	174%
NRG1 beta 1 (CON)	23%	22%	150%	158%
NT-3	5%	-2%	-9%	7%
NT-4	5%	7%	23%	6%
Oncostatin-M (209 a.a.)	15%	7%	150%	155%
Oncostatin-M (227 a.a.)	13%	10%	74%	105%
Osteoprotegerin	-7%	1%	-2%	-3%
OTOR	-2%	-4%	-5%	-3%
X40 Ligand (Insect cell derive	4%	1%	1%	-10%
PDGF	-26%	-26%	-43%	-48%
PDGF-AA	3%	1%	-7%	-12%
PDGF-AB	2%	0%	-2%	-5%
PDGF-BB	5%	2%	-10%	-5%
PDGF-CC	1%	2%	12%	-1%
ECAM-1 (HEK293 cell derived	-5%	-3%	5%	3%
PEDF	4%	4%	16%	12%
Persephin	0%	5%	30%	8%
PF-4 (CXCL4)	2%	5%	-8%	-1%
Pleiotrophin	2%	-3%	-5%	-12%
PlGF-1	-3%	-2%	0%	6%
PlGF-2	-1%	-2%	18%	1%
PlGF-3	-4%	0%	8%	11%
Prokineticin-2	2%	-3%	-7%	-5%
Prolactin	-9%	0%	-4%	-7%
PTHrP	-4%	-3%	-9%	-19%
RANTES (CCL5)	0%	-4%	-8%	-2%
Relaxin-2	-6%	0%	-8%	8%
Relaxin-3	-4%	-3%	19%	19%
RELM-beta	3%	6%	37%	9%
Resistin	-2%	4%	15%	-9%
sCD100 (CHO cell derived)	-1%	-4%	-3%	0%
sCD14 (293 cell derived)	-3%	-3%	-8%	-3%
sCD23	-2%	-1%	1%	4%
CD27 Ligand (CHO cell derivec	-34%	-38%	-82%	-94%
CD30 Ligand (CHO cell derivec	0%	4%	21%	17%
sCD34 (CHO cell derived)	0%	4%	12%	-1%
sCD40 Ligand	-6%	3%	0%	-9%
SCF	2%	1%	-12%	-11%

**SCGF-alpha**	9%	6%	21%	-6%
**SCGF-beta**	7%	3%	18%	-8%
**SDF-1alpha (CXCL12)**	3%	5%	-13%	-19%
**SDF-1beta (CXCL12)**	2%	-2%	-6%	-15%
**sDLL-1 (HEK293 cell derived)**	-2%	4%	3%	-15%
**sDLL-4 (HEK293 cell derived)**	-3%	-1%	-4%	1%
**sFas Ligand (CHO cell derived)**	4%	-3%	4%	-20%
**sFas Receptor**	-2%	-1%	-4%	-17%
**sFRP-1 (HeLa cell derived)**	-2%	-1%	1%	-14%
**IL-2Ralpha (Insect cell derived**	-1%	-4%	11%	-1%
**L-4Ralpha (HEK293 cell derive**	2%	-4%	-21%	-8%
**L-6Ralpha (HEK293 cell derive**	0%	-5%	-25%	-13%
**Slit2-N (HEK293 cell derived)**	2%	2%	3%	7%
**Sonic Hedgehog**	-2%	-3%	-4%	15%
**RC/Osteonectin (CHO cell deri**	-26%	-27%	-28%	-33%
**sRANK Ligand**	10%	-3%	-7%	-13%
**sRANK Receptor**	-8%	-3%	6%	-9%
**sTNF-receptor Type I**	-3%	0%	-8%	3%
**sTNF-receptor Type II**	-1%	-3%	-15%	-2%
**sTRAIL receptor-1**	-7%	-3%	-9%	-10%
**sTRAIL receptor-2**	-7%	-5%	-9%	3%
**sTRAIL/Apo2L**	-16%	-15%	-40%	-30%
**TACI**	4%	0%	-2%	-6%
**TAFA-2**	-1%	-5%	-2%	-4%
**TARC (CCL17)**	0%	6%	1%	10%
**TECK (CCL25)**	2%	2%	-10%	-11%
**TFF-1**	-1%	-1%	-4%	-5%
**TFF-2**	-4%	-5%	-13%	-9%
**TFF-3**	-4%	-2%	-8%	-13%
**TGF-alpha**	10%	17%	153%	163%
**TGF-beta 1**	-31%	-33%	-58%	-65%
**TGF-beta 2**	-21%	-19%	-37%	-34%
**TGF-beta 3**	-18%	-20%	-41%	-39%
**GF-beta1 (HEK293 cell derivec**	-7%	-16%	21%	11%
**GF-beta2 (HEK293 cell derivec**	-5%	-12%	-22%	-38%
**TGF-beta3**	-14%	-15%	-37%	-38%
**Thrombopoietin/Tpo**	-4%	1%	15%	3%
**TL-1A**	-2%	0%	2%	-19%
**TLR-3 (HEK293 cell derived)**	-4%	1%	12%	7%
**TNF-alpha**	-34%	-40%	-110%	-102%
**TNF-beta**	-28%	-32%	-54%	-50%

TPO	8%	1%	-34%	-27%
TSG	-8%	-2%	4%	0%
TSLP	10%	-3%	-16%	-1%
TWEAK	-8%	-4%	12%	27%
TWEAK Receptor	-3%	0%	-4%	-7%
VAP-1 (CHO cell derived)	6%	-3%	-7%	-11%
Vaspin	-2%	1%	6%	2%
VCAM-1 (HEK293 cell derived)	-3%	5%	29%	26%
VEGF-A (121 a.a.)	8%	-1%	-1%	-2%
VEGF-A (165 a.a.)	11%	5%	17%	20%
VEGF-B	-3%	-2%	19%	1%
VEGF-C (HEK293 cell derived)	-4%	-1%	25%	20%
VEGF-D (HEK293 cell derived)	8%	-1%	15%	-4%
Visfatin	7%	23%	115%	116%
WISP-1	0%	0%	-11%	-10%
WISP-3	1%	-3%	14%	3%
WNT-1	9%	-2%	-12%	-12%
Wnt-4	-24%	-24%	-36%	-43%
WNT-7A (HEK 293 cell derived	2%	-9%	-15%	1%
WISP-3	7%	1%	4%	17%
Angiogenin	-4%	-3%	-12%	-10%
AREG / GAS6	-6%	-5%	0%	-2%
BMP-10	-10%	-3%	-18%	-37%
BMP-5	6%	0%	1%	-10%
BOC	2%	-3%	-4%	-17%
Cathepsin S	12%	6%	15%	-5%
CCL11/Eotaxin	14%	11%	21%	16%
CCL13/MCP-4	2%	1%	22%	-4%
CCL14 (two aa forms) / CCL15	-11%	-6%	-21%	-5%
CCL15/MIP-1 delta	3%	2%	28%	-1%
CCL15/MIP-1 delta	4%	3%	6%	-11%
CCL16/HCC-4	-6%	2%	15%	-5%
CCL17/TARC	-4%	2%	7%	-8%
CCL19/MIP-3 beta	8%	0%	5%	-6%
CCL20/MIP-3 alpha	16%	14%	31%	6%
CCL21/6Ckine	1%	14%	21%	-10%
CCL22/MDC	5%	3%	24%	-7%
CCL23/Ck beta 8-1	3%	3%	27%	1%
CCL23/MPIF-1	6%	4%	14%	-2%
CCL24/Eotaxin-2/MPIF-2	0%	-1%	9%	-10%
CCL25/TECK	0%	1%	10%	-11%

CCL26/Eotaxin-3	8%	4%	-20%	-15%
CCL27/CTACK	18%	13%	24%	22%
CCL28	7%	9%	27%	-6%
L1/MIP-1 alpha Isoform LD78	6%	4%	17%	-8%
CCL4L1/LAG-1	1%	6%	24%	1%
CD30 Ligand/TNFSF8	13%	2%	30%	9%
CD40 Ligand/TNFSF5	3%	4%	8%	-5%
CTGF	-6%	-5%	-24%	0%
CX3CL1/Fractalkine	5%	3%	21%	-8%
CXCL11/I-TAC	0%	2%	26%	-1%
CXCL12/SDF-1	3%	5%	13%	-12%
CXCL13/BLC/BCA-1	10%	18%	50%	6%
CXCL14/BRAK	13%	15%	21%	-4%
CXCL17/VCC-1	1%	6%	17%	-6%
CXCL4/PF4	7%	8%	16%	-9%
CXCL5/ENA-70	5%	2%	10%	-14%
CXCL5/ENA-74	3%	10%	13%	7%
CXCL5/ENA-78	4%	4%	23%	1%
CXCL6/GCP-2	6%	4%	11%	-16%
CXCL9/MIG	0%	0%	11%	0%
Decorin	-9%	-5%	-17%	-2%
Desert Hedgehog/Dhh	-2%	6%	21%	-1%
Dkk-2	7%	3%	24%	-1%
Dkk-3	2%	1%	19%	4%
Dkk-4	4%	2%	17%	-1%
DPPIV	7%	5%	18%	4%
Draxin	19%	18%	6%	-8%
EGF	19%	15%	172%	114%
EGF (CON)	18%	26%	173%	124%
EGF (CON)	11%	6%	144%	121%
EGF (CON)	22%	19%	167%	101%
EGF / IL7 / NT3 / Eotaxin	7%	11%	130%	109%
EGF / NRG / FGFb / HGF	32%	30%	228%	167%
Endocan/ESM-1	-1%	3%	21%	1%
Ephrin-A1	1%	-1%	10%	-23%
Ephrin-A3	-2%	3%	-6%	-2%
Ephrin-A3	5%	5%	7%	-15%
Ephrin-A4	3%	-1%	0%	-13%
Ephrin-A4	15%	8%	8%	-6%
Ephrin-A5	-1%	8%	12%	-14%
Ephrin-A5	11%	11%	21%	4%

**Ephrin-B3**	6%	5%	10%	-3%
**Ephrin-B3**	8%	10%	11%	-7%
**ephrinB2**	-13%	0%	-3%	-8%
**EPO**	-9%	1%	-20%	-9%
**Fas Ligand/TNFSF6**	-8%	-1%	-1%	-4%
**FGF-12**	10%	5%	-4%	-5%
**FGF-22**	30%	24%	123%	81%
**FGF-3**	21%	26%	97%	71%
**FGF-acidic**	41%	37%	198%	148%
**FGF-basic**	30%	31%	177%	120%
**FGF-basic (CON)**	29%	35%	204%	136%
**FGF-basic (CON)**	12%	23%	154%	115%
**FGF-basic (CON)**	31%	33%	187%	135%
**FGF-BP**	9%	9%	9%	7%
**FGF1 / IL6**	27%	27%	174%	137%
**FGF2 / CCL3**	16%	22%	155%	129%
**FGF2 / CCL3 / CCL20**	24%	23%	157%	134%
**Fibulin 5/DANCE**	7%	10%	3%	7%
**FLRG**	-4%	-6%	1%	-6%
**Frizzled-1**	2%	3%	9%	-12%
**Frizzled-10**	-5%	2%	7%	-17%
**Frizzled-4**	2%	1%	18%	-15%
**Frizzled-5**	15%	10%	39%	13%
**Frizzled-5**	2%	9%	29%	-11%
**Frizzled-7**	3%	3%	23%	2%
**Frizzled-8**	-3%	6%	-4%	-8%
**Gas-6**	-6%	0%	20%	22%
**GDF-1**	22%	21%	100%	72%
**GDF-15**	-6%	-5%	-11%	-3%
**GDF-8/Myostatin**	4%	5%	10%	1%
**GFR alpha-3/GDNF R alpha-3**	3%	0%	19%	-3%
**GITR Ligand/TNFSF18**	9%	6%	4%	-8%
**Gremlin**	2%	6%	14%	-13%
**HGF (CON)**	18%	19%	137%	69%
**HGF (CON)**	-2%	-2%	3%	2%
**HGF (CON)**	8%	10%	21%	4%
**HGF / AREG**	-5%	-6%	2%	14%
**HGF / CCL28**	-4%	-10%	-9%	7%
**HGF / GAS6**	-7%	-10%	-8%	-12%
**IFN**	-22%	-16%	-27%	-48%
**IFN-alpha**	-12%	-15%	-54%	-44%

**IFN-alpha 1**	-8%	-4%	-1%	-17%
**IFN-alpha 2**	-10%	-13%	-33%	-43%
**IFN-alpha 4**	-1%	-5%	-12%	-20%
**IFN-alpha 4**	4%	-5%	-25%	-21%
**IFN-alpha A**	-18%	-21%	-57%	-47%
**IFN-alpha B2**	-14%	-13%	-58%	-50%
**IFN-alpha C**	-11%	-15%	-33%	-33%
**IFN-alpha D**	-5%	-5%	-6%	-11%
**IFN-alpha F**	-7%	-9%	-15%	-30%
**IFN-alpha G**	-9%	-9%	-15%	-29%
**IFN-alpha H2**	-20%	-18%	-59%	-49%
**IFN-alpha I**	-15%	-16%	-35%	-44%
**IFN-alpha J1**	5%	0%	0%	-21%
**IFN-alpha K**	-10%	-1%	-17%	-25%
**IFN-alpha WA**	-12%	-9%	-21%	-31%
**IL-1 alpha/IL-1F1**	-13%	-14%	21%	17%
**IL-17/IL-17A**	3%	1%	17%	2%
**IL-17C**	9%	5%	26%	-16%
**IL-18 / FGFBP1**	-5%	-4%	-11%	-11%
**IL-18/IL-1F4**	0%	-2%	8%	-2%
**IL-1F7/FIL1 zeta**	10%	5%	22%	4%
**IL-24**	17%	12%	41%	16%
**IL-26/AK155**	2%	4%	29%	-30%
**IL-26/AK155**	18%	13%	22%	-4%
**IL-27**	0%	2%	20%	-2%
**IL-28A/IFN-lambda 2**	7%	8%	20%	10%
**IL-28B/IFN-lambda 3**	7%	5%	16%	5%
**IL-29/IFN-lambda 1**	10%	7%	5%	0%
**IL-32 alpha**	-6%	4%	2%	-3%
**IL-36 alpha/IL-1F6**	8%	6%	23%	13%
**IL-36 beta/IL-1F8**	5%	5%	35%	3%
**IL-36 gamma/IL-1F9**	2%	8%	35%	4%
**6 / IL8 / GMCSF / CXCL1 / MCI**	-1%	1%	17%	9%
**IL8 / CXCL1 / CXCL2 / CXCL3**	-2%	3%	36%	25%
**Indian Hedgehog/Ihh**	0%	5%	19%	-14%
**Insulin**	-4%	-6%	24%	15%
**Klotho beta**	3%	0%	6%	-18%
**LEDGF**	13%	13%	32%	15%
**Lefty-A**	2%	2%	8%	-10%
**LIF**	0%	0%	15%	23%
**Media (+ DRUG)**	12%	11%	42%	16%

**Media (+ DRUG)**	-2%	-3%	-9%	4%
**Media (+ DRUG)**	7%	10%	40%	4%
**Media (+ DRUG)**	-9%	-7%	-16%	-8%
**Media (+ DRUG)**	-6%	-1%	-7%	-5%
**Media (+ DRUG)**	-7%	-7%	-9%	6%
**Media (+ DRUG)**	-6%	-5%	-16%	-8%
**Media (+ DRUG)**	-6%	-9%	-18%	-1%
**Media (+ DRUG)**	1%	5%	1%	2%
**Media (+ DRUG)**	0%	0%	-8%	3%
**Media (+ DRUG)**	-6%	-7%	-8%	7%
**Media (+ DRUG)**	-6%	-8%	-13%	-3%
**Media (+ DRUG)**	-9%	-5%	-9%	-6%
**Media (+ DRUG)**	-4%	-5%	-21%	-7%
**Media (+ DRUG)**	-7%	-9%	-13%	-11%
**Media (+ DRUG)**	-3%	-3%	4%	-2%
**Media (+ DRUG)**	1%	-1%	-12%	0%
**Media (+ DRUG)**	-4%	-1%	-18%	12%
**Media (+ DRUG)**	-1%	-3%	-6%	-7%
**Media (+ DRUG)**	-10%	-6%	-16%	1%
**Media (+ DRUG)**	-5%	-2%	-11%	-9%
**Media (+ DRUG)**	-6%	-10%	-5%	1%
**Media (+ DRUG)**	-5%	-9%	-7%	-7%
**Media (+ DRUG)**	-5%	-10%	-18%	-12%
**Media (+ DRUG)**	5%	-3%	-5%	1%
**Media (+ DRUG)**	4%	4%	-7%	-4%
**Media (+ DRUG)**	-1%	-3%	-5%	-3%
**Media (+ DRUG)**	-2%	-5%	-12%	1%
**Media (+ DRUG)**	-3%	-2%	-20%	-2%
**Media (+ DRUG)**	-3%	0%	-11%	5%
**Media (+ DRUG)**	-5%	-3%	-15%	1%
**Media (+ DRUG)**	1%	-5%	-15%	-1%
**Media (+ DRUG)**	17%	14%	17%	5%
**Media (+ DRUG)**	6%	6%	0%	-5%
**Media (+ DRUG)**	0%	14%	16%	-2%
**Media (+ DRUG)**	0%	5%	4%	-1%
**Media (+ DRUG)**	2%	6%	15%	-8%
**Media (+ DRUG)**	-6%	-2%	-15%	5%
**Media (+ DRUG)**	5%	2%	23%	0%
**Media (+ DRUG)**	-1%	-4%	-18%	5%
**Media (+ DRUG)**	10%	7%	9%	1%
**Media (+ DRUG)**	1%	-4%	-5%	-2%

**Media (+ DRUG)**	6%	10%	10%	-7%
**Media (+ DRUG)**	-2%	-4%	3%	0%
**Media (+ DRUG)**	3%	4%	17%	4%
**Media (+ DRUG)**	1%	-7%	-6%	3%
**Media (+ DRUG)**	11%	1%	32%	1%
**Media (+ DRUG)**	2%	1%	4%	7%
**Media (+ DRUG)**	7%	2%	9%	5%
**Media (+ DRUG)**	9%	7%	16%	-5%
**Media (+ DRUG)**	5%	0%	-2%	11%
**Media (+ DRUG)**	5%	5%	-15%	-14%
**Media (+ DRUG)**	-4%	1%	-11%	-6%
**Media (+ DRUG)**	-9%	-5%	-15%	-2%
**Media (+ DRUG)**	3%	-4%	-5%	5%
**Media (+ DRUG)**	-4%	-1%	-4%	-4%
**Media (+ DRUG)**	-7%	-5%	-5%	4%
**Media (+ DRUG)**	-11%	-1%	10%	13%
**Media (+ DRUG)**	9%	5%	29%	19%
**Media (+ DRUG)**	7%	12%	19%	-7%
**Media (+ DRUG)**	11%	8%	13%	-5%
**Media (+ DRUG)**	5%	6%	23%	-2%
**Media (+ DRUG)**	5%	5%	16%	8%
**Media (+ DRUG)**	-1%	5%	11%	-9%
**Media (+ DRUG)**	4%	4%	16%	2%
**Media (+ DRUG)**	0%	6%	28%	15%
**Media (NO DRUG)**	108%	130%	165%	86%
**Media (NO DRUG)**	92%	103%	86%	90%
**Media (NO DRUG)**	127%	136%	168%	91%
**Media (NO DRUG)**	111%	104%	128%	103%
**Media (NO DRUG)**	123%	132%	158%	103%
**Media (NO DRUG)**	104%	105%	125%	95%
**Media (NO DRUG)**	127%	125%	168%	90%
**Media (NO DRUG)**	109%	105%	128%	110%
**Media (NO DRUG)**	123%	123%	146%	88%
**Media (NO DRUG)**	100%	116%	122%	94%
**Media (NO DRUG)**	120%	121%	164%	104%
**Media (NO DRUG)**	95%	101%	109%	103%
**Media (NO DRUG)**	124%	125%	164%	102%
**Media (NO DRUG)**	103%	105%	114%	99%
**Media (NO DRUG)**	130%	124%	179%	100%
**Media (NO DRUG)**	111%	106%	89%	94%
**Media (NO DRUG)**	114%	128%	145%	95%

**Media (NO DRUG)**	103%	101%	90%	96%
**Media (NO DRUG)**	117%	125%	146%	89%
**Media (NO DRUG)**	106%	97%	86%	80%
**Media (NO DRUG)**	132%	124%	134%	95%
**Media (NO DRUG)**	107%	97%	76%	86%
**Media (NO DRUG)**	168%	161%	242%	164%
**Media (NO DRUG)**	95%	89%	89%	97%
**Media (NO DRUG)**	122%	120%	170%	92%
**Media (NO DRUG)**	85%	104%	80%	113%
**Media (NO DRUG)**	109%	128%	137%	84%
**Media (NO DRUG)**	97%	92%	82%	103%
**Media (NO DRUG)**	124%	112%	136%	102%
**Media (NO DRUG)**	90%	92%	76%	104%
**Media (NO DRUG)**	118%	111%	138%	85%
**Media (NO DRUG)**	102%	87%	96%	102%
**Media (NO DRUG)**	108%	105%	95%	102%
**Media (NO DRUG)**	104%	99%	82%	90%
**Media (NO DRUG)**	88%	90%	84%	93%
**Media (NO DRUG)**	95%	86%	79%	101%
**Media (NO DRUG)**	82%	97%	72%	94%
**Media (NO DRUG)**	90%	89%	85%	90%
**Media (NO DRUG)**	86%	96%	91%	82%
**Media (NO DRUG)**	89%	85%	91%	104%
**Media (NO DRUG)**	103%	108%	104%	116%
**Media (NO DRUG)**	97%	94%	80%	87%
**Media (NO DRUG)**	91%	93%	85%	83%
**Media (NO DRUG)**	91%	98%	84%	78%
**Media (NO DRUG)**	96%	89%	83%	87%
**Media (NO DRUG)**	83%	84%	89%	96%
**Media (NO DRUG)**	85%	90%	81%	93%
**Media (NO DRUG)**	77%	93%	96%	110%
**Media (NO DRUG)**	92%	94%	91%	109%
**Media (NO DRUG)**	101%	99%	84%	96%
**Media (NO DRUG)**	91%	102%	70%	101%
**Media (NO DRUG)**	86%	92%	93%	96%
**Media (NO DRUG)**	82%	87%	91%	90%
**Media (NO DRUG)**	84%	91%	84%	105%
**Media (NO DRUG)**	75%	87%	77%	81%
**Media (NO DRUG)**	90%	91%	86%	101%
**Media (NO DRUG)**	117%	98%	97%	123%
**Media (NO DRUG)**	98%	93%	100%	107%

**Media (NO DRUG)**	92%	93%	102%	79%
**Media (NO DRUG)**	88%	90%	87%	92%
**Media (NO DRUG)**	88%	77%	76%	89%
**Media (NO DRUG)**	91%	95%	62%	104%
**Media (NO DRUG)**	85%	88%	85%	93%
**Media (NO DRUG)**	104%	89%	101%	95%
**Media (NO DRUG)**	107%	100%	116%	113%
**Media (NO DRUG)**	100%	108%	112%	99%
**Media (NO DRUG)**	91%	94%	89%	95%
**Media (NO DRUG)**	93%	92%	80%	88%
**Media (NO DRUG)**	82%	88%	81%	79%
**Media (NO DRUG)**	78%	87%	62%	100%
**Media (NO DRUG)**	86%	89%	81%	89%
**Media (NO DRUG)**	93%	92%	94%	111%
**Media (NO DRUG)**	120%	109%	90%	125%
**Media (NO DRUG)**	100%	103%	75%	96%
**Media (NO DRUG)**	99%	88%	94%	93%
**Media (NO DRUG)**	81%	89%	91%	100%
**Media (NO DRUG)**	94%	80%	77%	92%
**Media (NO DRUG)**	92%	91%	97%	96%
**Media (NO DRUG)**	84%	86%	84%	101%
**Media (NO DRUG)**	90%	87%	89%	100%
**Media (NO DRUG)**	105%	111%	106%	119%
**Media (NO DRUG)**	100%	109%	95%	105%
**Media (NO DRUG)**	102%	100%	84%	108%
**Media (NO DRUG)**	104%	84%	72%	98%
**Media (NO DRUG)**	92%	92%	77%	105%
**Media (NO DRUG)**	93%	93%	89%	102%
**Media (NO DRUG)**	94%	84%	84%	103%
**Media (NO DRUG)**	102%	89%	97%	104%
**Media (NO DRUG)**	111%	110%	97%	108%
**Media (NO DRUG)**	106%	105%	83%	91%
**Media (NO DRUG)**	103%	93%	75%	94%
**Media (NO DRUG)**	90%	102%	87%	103%
**Media (NO DRUG)**	95%	87%	88%	93%
**Media (NO DRUG)**	95%	92%	85%	104%
**Media (NO DRUG)**	106%	89%	90%	84%
**Media (NO DRUG)**	106%	91%	94%	106%
**Media (NO DRUG)**	125%	98%	89%	108%
**Media (NO DRUG)**	106%	115%	95%	97%
**Media (NO DRUG)**	108%	104%	89%	93%

Media (NO DRUG)	100%	95%	78%	96%
Media (NO DRUG)	82%	93%	89%	97%
Media (NO DRUG)	104%	93%	97%	95%
Media (NO DRUG)	83%	93%	76%	107%
Media (NO DRUG)	101%	88%	83%	105%
Media (NO DRUG)	117%	109%	113%	104%
Media (NO DRUG)	97%	101%	108%	112%
Media (NO DRUG)	92%	104%	94%	105%
Media (NO DRUG)	89%	97%	98%	98%
Media (NO DRUG)	100%	92%	112%	102%
Media (NO DRUG)	107%	97%	94%	91%
Media (NO DRUG)	99%	93%	109%	98%
Media (NO DRUG)	89%	96%	99%	114%
Media (NO DRUG)	117%	106%	105%	117%
Media (NO DRUG)	119%	121%	104%	124%
Media (NO DRUG)	95%	102%	115%	123%
Media (NO DRUG)	84%	94%	110%	126%
Media (NO DRUG)	105%	94%	100%	118%
Media (NO DRUG)	86%	107%	103%	117%
Media (NO DRUG)	89%	107%	85%	125%
Media (NO DRUG)	85%	95%	97%	118%
MIS/AMH	2%	0%	25%	-7%
MMP-1	-1%	-1%	8%	1%
MMP-10	-4%	-5%	-17%	2%
MMP10 / ANG1	14%	12%	73%	59%
leuregulin-1 alpha/NRG1 alph	28%	32%	230%	152%
euregulin-1 beta 1/NRG1 beta	33%	26%	255%	168%
Neuregulin-1 Isoform SMDF	28%	30%	272%	186%
Neuregulin-1/NRG1	12%	17%	237%	151%
Neuropilin-1	10%	11%	31%	0%
Neuropilin-2	8%	4%	24%	1%
Nidogen-1	-9%	-5%	3%	-11%
Nodal	1%	2%	13%	-9%
NRG1 beta 1 (CON)	45%	46%	282%	193%
NRG1 beta 1 (CON)	15%	13%	222%	171%
NRG1 beta 1 (CON)	27%	26%	233%	152%
Oncostatin M/OSM	6%	8%	164%	101%
Osteoprotegerin/TNFRSF11B	8%	5%	10%	-5%
PAI-I	-6%	0%	-9%	3%
PDGF-DD	5%	2%	15%	-5%
Pentraxin 3/TSG-14	-6%	2%	10%	-6%

**PIGF**	-8%	-12%	-9%	15%
**Progranulin/PGRN**	-1%	2%	4%	-13%
**R-Spondin 1**	2%	2%	6%	-10%
**R-Spondin 2**	6%	2%	-21%	-16%
**R-Spondin 3**	7%	8%	24%	-3%
**R-Spondin 4**	3%	7%	29%	-6%
**RANK/TNFRSF11A**	-1%	3%	21%	-6%
**sFRP-3**	8%	14%	31%	2%
**sFRP-4**	3%	-1%	14%	-12%
**sFRP-5**	1%	-2%	11%	-1%
**Soggy-1/DkkL1**	4%	1%	25%	3%
**SOST/Sclerostin**	1%	3%	8%	-20%
**TAFA1/FAM19A1**	-3%	2%	-5%	-2%
**TAFA2/FAM19A2**	1%	4%	-2%	-17%
**TAFA4/FAM19A4**	9%	16%	13%	-1%
**TAFA5/FAM19A5**	9%	14%	2%	-6%
**Thrombospondin**	-7%	-3%	0%	-8%
**TIMP1**	4%	0%	-7%	9%
**TIMP2**	-8%	1%	0%	3%
**TIMP2 / Osteoprotegerin**	-6%	-7%	-9%	-16%
**TIMP2 / SPARC**	-5%	-8%	-7%	-7%
**TL1A/TNFSF15**	12%	13%	55%	31%
**TRAIL/TNFSF10**	-17%	-17%	-69%	-66%
**TRANCE/TNFSF11/RANK L**	3%	2%	27%	-3%
**TROY/TNFRSF19**	6%	3%	-1%	2%
**uPAR**	-8%	-5%	-16%	-7%
**VEGF**	-1%	3%	5%	-7%
**VIP**	-4%	-4%	0%	-6%
**WIF-1**	1%	7%	33%	-11%
**WISP1**	-3%	1%	-15%	-2%
**Wnt-11**	-1%	2%	12%	-4%
**Wnt-3a**	-1%	1%	13%	-11%
**Wnt-5a**	3%	2%	17%	-10%
**XCL1/Lymphotactin**	-3%	-1%	-4%	-13%
